# Teaching medical students remotely during a pandemic – what can psychiatry offer?

**DOI:** 10.1177/1039856220971931

**Published:** 2020-11-24

**Authors:** Taraneh Khoo, Nicola Warren, Anna Jenkins, Jane Turner

**Affiliations:** The University of Queensland Faculty of Medicine and Biomedical Sciences, Herston, QLD, Australia; Royal Brisbane and Women’s Hospital, Herston, QLD, Australia; The University of Queensland Faculty of Medicine and Biomedical Sciences, Herston, QLD, Australia; Metro South Health Service District, Addiction and Mental Health, Woolloongabba, QLD, Australia; The University of Queensland Faculty of Medicine and Biomedical Sciences, Herston, QLD, Australia; Royal Brisbane and Women’s Hospital, Herston, QLD, Australia; The University of Queensland Faculty of Medicine and Biomedical Sciences, Herston, QLD, Australia; Royal Brisbane and Women’s Hospital, Herston, QLD, Australia

**Keywords:** teaching, psychiatry, medical students, pandemic, online

## Abstract

**Objective:**

The clinical teaching of psychiatry to medical students throughout the COVID-19 pandemic has presented opportunities for support, engagement and learning above and beyond usual practice. Like other teaching faculties, we needed to quickly adapt the course material to an online platform. However, for psychiatric teaching, it was also essential to find alternatives to patient interviewing, and to provide support and containment in uncertain times. We aim to describe our philosophical stance and framework for the delivery of our online course.

**Conclusions:**

Key components in the delivery of our modified course were the transition to online learning and assessment, developing a suite of surrogate clinical learning experiences, using simulated patients for online interviewing, and attention to student well-being whilst providing a supportive and contained environment for student learning. Supportive leadership and good communication assisted the teaching staff to deliver the course during COVID-19.

Teaching psychiatry to medical students during COVID-19 required the rapid adaptation of the existing course into an online platform.^1^ The increasing use of active learning techniques even before the pandemic meant that there was some degree of preparation to transition to a fully online teaching programme.^2,3^ However, other aspects of the course such as ward exposure and facilitated clinical interviewing could not be captured by direct transfer into didactic lectures. Novel strategies were required.

During disasters or crises, a range of psychological symptoms may appear in health professionals.^4^ Medical students may have a pre-existing vulnerability, with higher levels of psychological distress often reported during clinical practice years.^5^ International students face additional challenges of living away from family members. Student welfare and well-being needed to be prioritised at this time.^6^

The University of Queensland’s psychiatric teaching staff aimed to provide not just a substitute for the usual course, but rather a comprehensive, enriched online course as well as a supportive and contained environment for the students. Table 1 outlines the characteristics of the students taught during COVID-19. The following is an outline of the modifications that were made to teach psychiatry remotely during the pandemic.

**Table 1.** Characteristics of the psychiatry teaching course during COVID-19

Location	University of Queensland
Subject	Psychiatry
Year level	Third year medicine
Number of students	
• domestic	45
• international	18
Total	63
Placement length	6 weeks
Number of teaching staff	four part-time teaching clinicians (1.2 FTE*)

*Note.* *FTE = full-time equivalents.

## Transitioning to complete online learning and assessment

Existing lectures and interactive online cases using Articulate^7^ and Blackboard^8^ were supplemented with recorded clinical interviewing techniques, mental state assessments and formulation presentations to replace face-to-face teaching. A modified curriculum was developed to take into account missed ward exposure. Multiple articles and resources were provided online, and to facilitate critical appraisal of the material, resources were linked to associated content with annotated notes or prompt sheets.^9^ Students were able to access this material throughout their placement and semester.

Videoconferencing using Zoom^10^ provided group learning and discussion. Students were placed in small groups (maximum of eight) to encourage greater dialogue. Teaching staff expected the small groups to demonstrate a high level of engagement, as had previously been found when teaching small groups face to face. However, the group dynamics in online sessions were different – students needed more encouragement, and sessions were draining for the moderators, perhaps because of the communication barriers associated with the technology.^11^

The usual written and viva psychiatry examinations were adapted to an online platform using simulated patients and software with invigilation.^12^ The viva component was replaced with a brief observed interview with a simulated patient via Zoom videoconferencing. It was not feasible to offer real patients for the viva given the student numbers. The written examinations consisted of (a) a 60-item multiple-choice questionnaire, and (b) a long answer case requiring a formulation and management plan of a hypothetical patient. Challenges with these examinations included difficulties with the internet and technology, and privacy concerns as invigilators could see into students’ homes. Pleasingly, performance on end-of-semester written examinations was found to be comparable to previous placements.

## Provision of structure

Suspended hospital placements meant there was a loss of the usual structure and daily routine. Additional sessions were provided which included a weekly wrap-up online to consolidate learning. A structured daily timetable assisted with time management, with time allotments for each task and links to associated resources. No tasks were assigned to afterhours or weekends promoting a clear demarcation of working hours. Senior staff prioritised self-care during online teaching sessions.

## Surrogates for patient interviewing

Access to patients via teleconferencing was not feasible due to the lack of videoconferencing facilities on the ward and the pressures on the ward clinicians during the pandemic. In the absence of ward exposure, a rapid substitute for teaching psychiatric interviewing skills was required. Videos of clinicians conducting patient interviews were uploaded to Blackboard. The use of simulated patients in medicine has become widespread as it allows students to practice skills in a low-risk environment without the fear of distressing the patient.^13^ Clinicians engaged in role-play with students to simulate patient interviewing. Immediate feedback was given, and students could attempt the interview several times to shape their skills. However, on review, it was felt this was an insufficient substitute and instead we chose to employ a trained simulated patient. A detailed script was developed to ensure consistency, and the sessions were moderated by a senior psychiatrist.

## Ensuring student engagement, welfare, containment and support

As traditional pastoral care was not possible during COVID-19, alternatives were required to assist students facing social isolation.^14^ Techniques that can reduce psychological distress during disasters, such as limiting exposure to media, sleep hygiene, exercise and nutrition, and the resumption of normal routines and roles, were incorporated into online discussions.^15^ Resources about self-care were provided to promote well-being and improve adaptation to adversity. A closed social media group was set up to provide dynamic, synchronous communication.^16^ This replaced the pre-COVID-19 drop-in style sessions where students could ask questions, clarify concerns, query timetable changes or make comments. Staff provided information and posted links to both clinical and well-being resources. The group was monitored to ensure appropriate interactions.

Students were provided with access to reduced cost psychiatric care and pathways to access mental health services. Staff were able to provide remedial tutorials and referral to appropriate agencies if required.

Careful attention was given to the tone of online interactions, with the aim of promoting collegiality and modelling authentic engagement. Borrowing from the work of Bion, clinicians focussed on providing containment in teaching sessions and allowing for the identification and processing of projected feelings from the students.^17^ These feelings could then be returned to students in a modified way that could be reintegrated. Sessions were deliberately less formal. Distress and uncertainty were acknowledged, expressed and validated by using praise, guiding self-care and emotional mirroring and validation (see Table 2). Students responded positively to the social media group in feedback given at the end of placement evaluations.

**Table 2.** Examples of support provided by teaching staff on social media

Guiding self-care	*“Hope everyone has had a gentle start to swot vac. Important to maintain a routine and include plenty of breaks and exercise. Remember that all rules about avoiding excess treats do not apply as they are considered essential in swot vac.”*
Praise to reinforce engagement	*“During this placement I have been impressed by your enthusiasm, good grace and flexibility. You have demonstrated amazing learning and a level of sustained interest and curiosity which has made the past 6 weeks an absolute pleasure for me as a teacher.”*
Emotional mirroring and validation	*“You are right, these are uncertain times, and I am worried too”.*

## Promotion and advocacy of psychiatry

An integral part of teaching psychiatry is addressing and reducing stigma. Relevant TED talks and documentaries were provided to encourage discussion and provide exposure to those with lived experience with mental illness.

Videoconferencing allowed increased opportunities to provide additional sub-specialty teaching with expert staff in forensics and eating disorders. Numbers were capped at eight students to promote discussion, and these sessions proved to be one of the most popular changes to the course. High achieving students were catered for with the provision of additional resources that were not required reading but that offered an extension of knowledge.

## What challenges were identified in the move to online teaching?

The move to a fully online psychiatry course until restrictions lifted was not without difficulties. When restrictions eased, face-to-face teaching and ward exposure resumed in a limited form in line with hospital requirements. Students remained able to access the online resources. A short supplementary placement (5 weeks) was arranged at the end of the year for students affected by the lack of patient contact. The placement focused on improving interviewing skills. Modifying the course to an online platform took considerable effort, especially with the development of new content, timetables and linked resources. The move to multiple small group sessions was time intensive. Clinicians were not exempt from emotional strain during the pandemic. Weekly teleconferenced debriefings were instituted to prevent staff burnout. Teaching staff relied on the advice and encouragement from the course coordinator who was readily available for advice and support. Novel teaching approaches such as the use of social media were welcomed. Teaching and administrative staff learnt to use new technology in the absence of infrastructure support. Internet and bandwidth issues were common. The costs of technology were significant for licenced software. See Table 3.

**Table 3.** Benefits and obstacles in delivering the online psychiatry teaching course

	Benefits	Obstacles
Course factors	• Pre-existing online material facilitated the move to remote learning • Didactic teaching converted well to small group teaching or online resources • Simulated patients provided interviewing experience	• Lack of ward exposure • Unable to provide large group teaching • Development of new resources was time consuming • Simulation could not fully replace actual patient contact • Increased burden on teaching and administrative staff • Unable to assess clinical skills
Student factors	• Able to learn from home • Familiar with the technology • Independent learners • Avoids exposure to contagion	• Concerns about clinical skill acquisition • Loss of daily structure was destabilising • Lack of social contact • Variable engagement with online group work • Tedium of extended online learning • Concerns about academic progression • Privacy concerns when online and with examination invigilation
Lecturer factors	• Able to work from home • Improved team cohesion as staff worked together on providing the modified course • Good leadership ensured support and assistance	• Online sessions required more cognitive load, perhaps because of the lack of non-verbal cues • Need to upskill rapidly in technological platforms • Risk of burnout with back-to-back online teaching sessions • Privacy concerns when online as students could see into homes
Technology factors	• Catered to the diverse learner • Interactive cases provided a novel learning experience • Information could be accessed at any time and rate • Social media facilitated communication and provided support	• Internet issues • Cost of licenses for interactive cases • Loss of non-verbal cues • Delays in sound/picture made group sessions challenging • Privacy issues with examination software that used invigilation

## Conclusion

As the world comes to terms with COVID-19, it seems clear that remote teaching will continue to play a significant role in medical education and innovative ways to provide surrogates for clinical contact will need to be found. Attention to student welfare and containment of psychological distress will continue to be an important role for educators. Remote learning is improving rapidly; however, it is unclear how it compares to direct ward teaching for specialties such as psychiatry. In particular, interviewing skills are more difficult to teach without face-to-face patient contact. Alternative methods to teach medicine will need to be explored, such as using social media to maintain connectivity and virtual ward rounds.

Delivering a fully online course was costly, time intensive and did not translate well to large class sizes. The loss of social interaction for students may have longer term impacts, necessitating ongoing adaptation and consideration of how best to encourage social cohesion and support networks in novel learning environments. Psychiatrists are in a unique position as medical specialists with a deep understanding of the human condition and specialised knowledge of the ways in which people respond to and adapt to stressful environments. Utilisation of such knowledge can optimise the way we teach our future health professionals as we adapt to these challenging times.

## References

[1] Torda AJ Velan G Perkovic V . The Impact of COVID-19 pandemic on medical education. Med J Aust (in press).

[2] Baepler P Walker J Brooks D , et al. A guide to teaching in the active learning classroom: history, research, and practice. Sterling, VA: Stylus Publishing, 2016.

[3] Warren N Parker S Khoo T , et al. Challenges and solutions when developing online interactive psychiatric education. Australas Psychiatry 2020; 28: 359–362. 32019351 10.1177/1039856220901477

[4] O’Byrne L Gavin B McNicholas F . Medical students and COVID-19: the need for pandemic preparedness. J Med Ethics 2020; 46: 623–626. 32493713 10.1136/medethics-2020-106353 PMC7316103

[5] Dendle C Baulch J Pellicano R , et al. Medical student psychological distress and academic performance. Med Teach 2018; 40: 1257–1263. 29355074 10.1080/0142159X.2018.1427222

[6] Guerandel A McCarthy N McCarthy J , et al. An approach to teaching psychiatry to medical students in the time of Covid-19. Ir J Psychol Med 2020; 1–19. 10.1017/ipm.2020.87 PMC7463153 32611461

[7] Articulate Storyline 360 computer software, 2018.

[8] Blackboard, Blackboard Learn TM, 2009.

[9] Abiodun RC . Annotation for knowledge sharing in a collaborative environment. J Knowl Manag 2009; 13: 111–119.

[10] Zoom Video Communications Inc, 2016.

[11] Kebritchi M Lipschuetz A Santiague L . Issues and challenges for teaching successful online courses in higher education: a literature review. J Educ Tech Syst 2017; 46: 4–29.

[12] Examplify, TM ExamSoft Worldwide Inc, 2020.

[13] Eagles J Calder S Wilson S , et al. Simulated patients in undergraduate education in psychiatry. Psychiatr Bull 2007; 31: 187–190.

[14] Hodgson J Hagan P . Medical education adaptations during a pandemic: transitioning to virtual student support. Med Educ 2020; 54: 662–663. 32291794 10.1111/medu.14177

[15] Kisely S Warren N McMahon L , et al. Occurrence, prevention, and management of the psychological effects of emerging virus outbreaks on healthcare workers: rapid review and meta-analysis. Br Med J (Clinical Research Ed.) 2020; 369: m1642. 10.1136/bmj.m1642 PMC7199468 32371466

[16] Walsh K . Synchronous telecommunications in medical education. J Biomed Res 2016; 30: 79–80. 26679786 10.7555/JBR.30.20150094 PMC4726838

[17] Bion W. ProQuest. Learning from experience. London: Karnac Books, 1984.

